# Sodium Balance and Quality of Life in People with Chronic Kidney Disease—A Cross-Sectional Study

**DOI:** 10.3390/nu17162634

**Published:** 2025-08-14

**Authors:** Kylie Martin, Sven-Jean Tan, Timothy D. Hewitson, Nigel D. Toussaint

**Affiliations:** 1 Department of Nephrology, The Royal Melbourne Hospital, Parkville, Melbourne, VIC 3050, Australia; jean.tan@mh.org.au (S.-J.T.); tim.hewitson@mh.org.au (T.D.H.); nigel.toussaint@mh.org.au (N.D.T.); 2 Department of Medicine (RMH), University of Melbourne, Melbourne, VIC 3010, Australia

**Keywords:** chronic kidney disease, quality-of-life, sodium

## Abstract

**Background:** Dietary sodium restriction in people with chronic kidney disease (CKD) may improve blood pressure and cardiovascular outcomes. However, little is known about body sodium levels (and dietary sodium restriction) on patient-reported health-related quality of life (HRQOL) in CKD. The primary aim of this study was to assess potential relationships between routinely used sodium measurements in clinical practice and acceptance of diet and fluid restrictions with HRQOL outcomes in people with CKD. **Methods:** We conducted a cross-sectional pilot study in 53 people with CKD, including those on dialysis, to explore relationships between HRQOL outcomes using the Kidney Disease Quality of Life-36 (KDQOL-36) questionnaire and measures of dietary sodium intake and urinary sodium excretion. **Results:** Participants with low 24 h urinary sodium excretion reported poorer HRQOL in kidney-specific domains with lower KDQOL-36 component scores for effects of kidney disease (*p* = 0.03) and a trend towards lower scores in burden of kidney disease and symptoms of kidney disease (both *p* = 0.06). Those who had lower acceptance of fluid and diet restriction had poorer HRQOL in kidney-specific domains with lower KDQOL-36 component scores for burden of kidney disease and effects of kidney disease (all *p* ≤ 0.01). **Conclusions:** Low 24 h urinary sodium excretion and lower acceptance of fluid and diet restriction in people with CKD are associated with poorer HRQOL scores in domains that assess level of kidney disease interference with life. Further studies exploring underlying mechanisms between urinary sodium excretion and HRQOL in CKD are needed. Efforts to increase acceptance of diet and fluid restrictions in people with CKD may improve HRQOL outcomes.

## 1. Introduction

Excess intake of dietary salt (sodium chloride) is a leading but modifiable risk factor in high blood pressure [1]. Globally, just over half of adults with hypertension are diagnosed, only 42% are receiving treatment, and only 21% have achieved their blood pressure goal [2]. Sodium exerts effects on blood pressure and contributes to cardiovascular, cerebrovascular, and kidney disease [3]. This occurs via multiple pathways including extracellular volume expansion (in the intravascular space), via tissue interstitial storage (such as in skin and muscle), and through disruption of inflammation and metabolic profiles [4,5].

Body sodium is increased in people with chronic kidney disease (CKD) and the negative impact of sodium on health outcomes is intensified in this cohort. The kidneys have a decreased ability to excrete sodium with progressive kidney function decline [6]. Increased circulating aldosterone levels [7], activation of the intrarenal renin–angiotensin system [7] and metabolic acidosis also stimulate sodium reabsorption [8]. Sodium is also implicated in uremia-related changes in metabolism and muscle mass [9], influencing oxidative stress pathways leading to inflammation, endothelial cell injury, and proteinuria [10,11]. These high salt diet-driven metabolic consequences can lead to sarcopenia with associated insulin resistance and obesity, accentuated in people with CKD [12].

As the prevalence of CKD continues to rise globally [13], improving the management of sodium imbalance is particularly important to mitigate adverse health outcomes in the CKD population. Guidelines recommend adults with CKD limit sodium intake to less than 2–2.3 g/day [14,15] but sodium consumption in most countries exceed this target [16]. Lowering sodium consumption in Australia could deliver substantial CKD health and economic benefits [17].

Furthermore, people with CKD, especially those on dialysis, carry a substantial physiological and psychological symptom burden, leading to poor health-related quality of life (HRQOL) [18]. Promoting better HRQOL is increasingly recognized for its role in improving patient satisfaction and engagement with healthcare [19]; and poor HRQOL is associated with adverse clinical outcomes, including cardiovascular events, CKD progression, and death [20,21,22].

Although many studies have emphasized the considerable symptom burden and poor HRQOL in people with CKD [18], little is known about the interaction between body sodium levels (evaluated by dietary intake and urinary excretion) and patient acceptance of diet and fluid restrictions on overall HRQOL in people across the spectrum of CKD, including those on dialysis. A tele-counseling application program with a dietician was reported in one study to improve sodium intake and blood pressure parameters [23] but there was no assessment on HRQOL. Another study that implemented a self-managed sodium restriction program (comprising education, motivational interviewing, coaching, and self-monitoring of blood pressure and sodium) demonstrated improvement in sodium measures and office blood pressure readings but with no change in HRQOL [24].

Management of body sodium balance with diet and fluid restriction is central to clinical care and HRQOL of people with CKD, but more research is needed to explore potential relationships between these factors. To address the current literature gap, the aims of this small, cross-sectional study were to assess relationships between HRQOL outcomes, participants’ acceptance of diet and fluid restriction, and routinely used body sodium measurements used in clinical practice for people with CKD.

## 2. Materials and Methods

### 2.1. Design

This small cross-sectional study assessed associations between sodium measurements and HRQOL outcomes in people with CKD, including those on hemodialysis (HD) and peritoneal dialysis (PD), in an Australian setting.

### 2.2. Participants and Procedures

Study participants were recruited from The Royal Melbourne Hospital Nephrology Outpatient Department between November 2022 and July 2023. Study participants were ≥18 years of age and provided written informed consent. Participants were recruited into three cohorts: (i) people with CKD stages 3–5 not on dialysis (according to the Kidney Disease: Improving Global Outcomes [KDIGO] guidelines [25]), (ii) those on chronic thrice-weekly HD, and (iii) those on PD. Both HD and PD patients had been established on their respective dialysis modality for at least 3 months.

### 2.3. Assessment of Quality of Life and Symptoms

HRQOL was assessed using the Kidney Disease Quality of Life (KDQOL)-36 instrument [26] where higher scores indicate better HRQOL and differences in more than 3.0 points are considered clinically significant [27]. This includes two composite HRQOL scales from the short form (SF)-12 version, namely the mental component score (MCS) and physical component score (PCS) [26], derived from the Medical Outcomes Study SF-12 (a generic HRQOL survey instrument used in healthy individuals and across many disease states [28]). The KDQOL-36 was used to assess kidney disease-targeted domains with component scores for “symptoms of kidney disease”, “burden of kidney disease”, and “effects of kidney disease”. Scales were scored by transforming all items linearly on a 100-point scale and averaging items in the scale [26]. A paper KDQOL-36 form was self-completed by participants after verbal instructions from study personnel, unless reading or comprehension problems precluded self-administration, in which case study personnel assisted participants in form completion.

People with CKD are routinely provided with dietary advice and advised of the importance of fluid management by their clinicians. People with CKD may be prescribed diet and fluid restrictions by their clinicians or engage in self-regulation of diet and fluid intake as part of their CKD management. As part of the KDQOL-36, we assessed patient acceptance of diet and fluid restrictions. We recorded on a Likert scale how bothered participants were by their restrictions where 1 = not at all bothered, 2 = somewhat bothered, 3 = moderately bothered, 4 = very much bothered, and 5 = extremely bothered. These five possible responses were collapsed into two categories, where not or somewhat bothered was classified as having “higher level of acceptance” of the restriction, and moderately to extremely bothered was classified as having a “lower level of acceptance” as previously used [29]. The component score “effects of kidney disease” includes an item to assess how bothered participants were by their diet and fluid restriction. In assessment of high vs. low acceptance of dietary and fluid restriction, the scores for how bothered participants were by their “diet restriction” and “fluid restriction” were removed from the component score “effects of kidney disease”.

### 2.4. Assessment of Sodium Intake and Excretion

To assess dietary sodium, we conducted the following: (a) a scored sodium questionnaire (SSQ) that has been validated in the Australian CKD population [30]; and (b) a 24 h dietary food record. For the SSQ, participants scored points on frequency and type of sodium-containing food intake (total of 215 points where larger values indicate higher sodium consumption and where ≥65 points indicates a high sodium consumer). For the 24 h dietary record, participants were prompted to include an estimation of amount or weight of foods consumed (e.g., as cups, handful, number). Participants were asked if there were any additional snacks or fluid consumption, and to estimate this quantity. Twenty-four-hour dietary sodium quantification was calculated using Foodworks Online [31]. Foodworks Online calculates sodium in foods from AusFoods (an expanded set of food descriptions with nutrient data from AUSNUT 2011-13 and the new Australian Food Composition Database) and AusBrands (contains nutrient data for commercial food products sold in Australia) [32]. Twenty-four hour and spot urine sodium collections were used to assess sodium excretion. The 24 h urinary sodium excretion (in mg/day) was calculated by the product of 24 h urinary sodium and urinary volume by 23 (molecular weight of sodium). The 24 h urine samples and spot urine tests were conducted in the same 24 h period as the dietary record in all but 5 participants (3 people with CKD and 2 people on PD who conducted the tests within 3 months of completing the KDQOL-36).

### 2.5. Outcomes and Statistical Analysis

Statistical analysis was performed using SPSS for Mac version 30.0 (IBM, Armonk, NY, USA). Parametric distribution was assessed both graphically and with the Shapiro–Wilk test. Continuous variables were presented as mean ± standard deviation (SD) if normally distributed and median [interquartile range (IQR)] if non-normally distributed. Categorical variables were expressed as proportions and percentages. All available data variables were analyzed, and no participant was removed. Differences between CKD groups were determined using one-way ANOVA, Kruskal–Wallis, and Chi-squared test or Fisher’s exact test for normally distributed continuous, non-normally distributed continuous, and categorical variables, respectively. Differences between groups of fluid and diet restriction acceptance were determined using independent-samples *t*-test and Mann–Whitney U test for normally distributed and non-normally distributed continuous variables, respectively. Ninety-five percent confidence intervals were computed and *p* < 0.05 was considered statistically significant.

## 3. Results

### 3.1. Baseline Characteristics and Medications

Data for a total of 53 participants (28 people with CKD, 13 on HD, and 12 on PD) were collected (Table 1). The CKD group consisted of patients with Stage 3a (*n* = 3), Stage 3b (*n* = 11), Stage 4 (*n* = 9), and Stage 5 (*n* = 5) CKD. Median age, sex, and body mass index distribution were comparable between the CKD, HD, and PD groups. There was no difference in prescribed medications between the three groups. Participants were considered anuric if they self-estimated their urinary volume < 200 mL/day if on HD (*n* = 5) or collected < 200 mL/day on a 24 h urine collection if on PD (*n* = 1); with two people with CKD, one person on PD, and two people on HD opting not to undertake a 24 h urine collection. For people on HD, mean dialysate sodium was 137.9 ± 2.0 mmol/L, median ultrafiltration rate was 480 mL/h [IQR 370–639], and median interdialytic weight gain was 1500 g [850–2000]. Of the 12 people on PD, 1 was on continuous ambulatory PD and 11 on automated PD. Glucose-based and icodextrin-based PD solutions contained a sodium concentration of 132 mmol/L. Peritoneal dialysis patients had a median total dwell volume of 6480 mL/day [4012.5–8648.75] and a mean ultrafiltration volume of 308.3 ± 267 mL/day. For people on PD, total median Kt/V was 2.14 [1.76–2.52] and residual kidney function median Kt/V was 1.25 [0.91–1.56].

Table 2 shows sodium measurements using routinely used measures of dietary intake and urinary excretion in clinical practice.

### 3.2. Associations Between Sodium Measurements and HRQOL

Table 3 shows KDQOL-36 composite and component scores for each group of CKD, HD, and PD. There were differences between participants with CKD, those on HD, and those on PD for KDQOL-36 components of burden of kidney disease (*p* = 0.03) and effects of kidney disease (*p* = 0.05). Those with CKD (*n* = 28) compared to those on dialysis (*n* = 25) had better HRQOL indicated by higher KDQOL-36 component scores for burden of kidney disease (*p* = 0.01) and effects of kidney disease (*p* = 0.02). Further evaluation of the CKD cohort separated into Stage 3a, 3b, 4, or 5 CKD, or comparing those on diuretics vs. not, and on sodium-glucose co-transporter 2-inhibitors (SGLT2-i) vs. not, showed no difference in kidney disease specific component scores but numbers were small in each group.

Table 4A–C show median scores for each KDQOL-36 component scores stratified by high and low sodium consumers defined by dietary SSQ (<65 vs. ≥65 points, respectively), 24 h dietary record (<2.3 g/day vs. ≥2.3 g/day, respectively), and high vs. low 24 h urinary sodium excretion (<2.3 g/day vs. ≥2.3 g/day, respectively), respectively. No difference was seen in KDQOL-36 component scores stratified by high vs. low sodium intake as per SSQ or 24 h dietary recall (Table 4A,B). However, participants with low 24 h urinary sodium excretion (<2.3 g/day) had lower KDQOL-36 component scores for effects of kidney disease (*p* = 0.03) and a trend towards lower scores in burden of kidney disease (*p* = 0.06) and symptoms of kidney disease (*p* = 0.06) (Table 4C). This indicates low 24 h urinary sodium excretion was associated with poorer HRQOL in these kidney-specific domains.

Analysis of HRQOL outcomes stratified by acceptance of diet and fluid restriction was undertaken. Of a total of 53 participants, 40 (75.5%) were classified as having high acceptance of their dietary restriction, and 43 (81.1%) as having high acceptance of their fluid restriction. Table 5A shows median scores for each KDQOL-36 component score according to the level of acceptance of diet restriction. Those who had lower acceptance of diet restriction (classified as moderately to extremely bothered by diet restriction), compared to patients who had higher acceptance of fluid restriction (classified as not at all or somewhat bothered by diet restriction), had lower scores in burden of kidney disease and effects of kidney disease (both *p* ≤ 0.01). Table 5B shows median scores for each KDQOL-36 component according to the level of acceptance of fluid restriction. Similarly, those who had lower acceptance of fluid restriction (classified as moderately to extremely bothered by fluid restriction), compared to patients who had higher acceptance of fluid restriction (classified as not at all or somewhat bothered by fluid restriction), had lower scores in burden of kidney disease and effects of kidney disease (both *p* ≤ 0.01).

**Figure 1:**
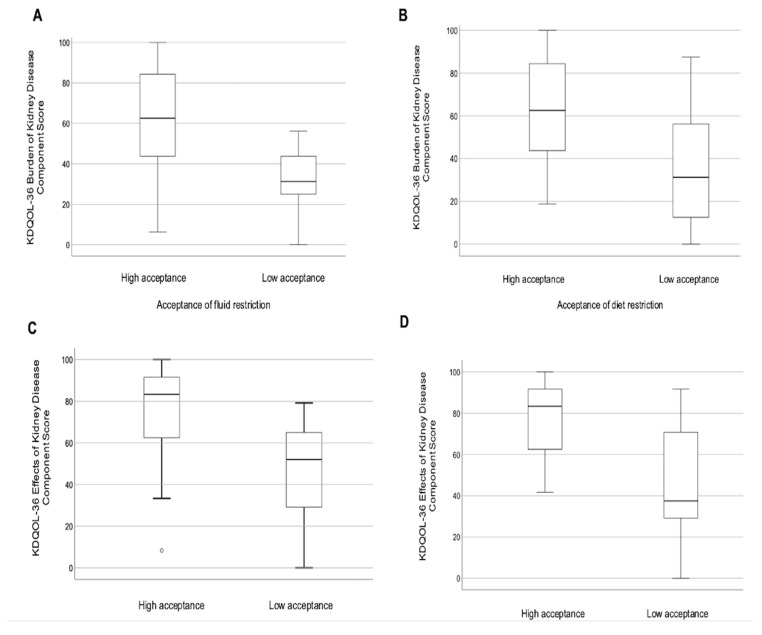
Boxplots of KDQOL-36 kidney-specific component scores for burden of kidney disease stratified by (**A**) acceptance of fluid restriction and (**B**) acceptance of diet restriction; and for effects of kidney disease stratified by (**C**) acceptance of fluid restriction and (**D**) acceptance of diet restriction.

These results indicate that those less accepting of their diet and fluid restrictions felt their kidney disease interfered more with, or represented more burden in, their lives (Figure 1). Further analysis showed there was no difference in kidney-specific HRQOL domains or level of fluid and diet restriction acceptance between those who were anuric compared to those on HD who collected a 24 h urine volume > 200 mL/day.

## 4. Discussion

We report, to our knowledge, the first study exploring relationships between body sodium measurements and prescribed diet and fluid restrictions, with HRQOL outcomes across the spectrum of CKD, including those on dialysis. Those with low urinary sodium excretion of <2.3 g sodium/day demonstrated poorer HRQOL (lower component scores for effects of kidney disease, and a trend towards lower scores in burden and symptoms of kidney disease). These findings are consistent with a large Korean cross-sectional study, which reported low estimated 24 h urine sodium associated with poorer HRQOL, even after adjusting for CKD [33]. This could be explained by inadequate nutrition or activation of poor metabolic profiles in people with low urinary sodium excretion (reflective of lower sodium dietary intake) [33].

Lack of differences in kidney specific HRQOL scores when participants were stratified based on two different measurements of dietary sodium intake (SSQ and 24 h dietary record) may be explained by the inherent difficulty in quantifying body sodium in those with CKD. Although 24 h urine sodium excretion is considered the gold-standard method to estimate dietary sodium intake, people with CKD have disrupted sodium physiology with decreased urinary sodium excretion [4] and increased sodium tissue storage [5]. Food frequency questionnaires and 24 h dietary records are prone to recall bias and are subject to considerable daily variation in food intake [4]. Several possible reasons why low urinary sodium excretion in our cohort was associated with poorer HRQOL parameters include the following: (i) greater restricted sodium and fluid intake that impacts the psychosocial dietary experience (supported by our finding of those with low 24 h urinary sodium excretion (<2.3 g/day) being less accepting of their fluid restriction); (ii) a higher burden of medications and medical therapies that alter urinary sodium excretory capacity, e.g., diuretics and SGLT2-i, have variable natriuretic effects [34,35]; and/or (iii) increased sodium storage in the tissue interstitial compartments of people with CKD which has been shown to be associated with adverse clinical outcomes [5].

Our study found those with lower acceptance of fluid and diet restriction have poorer HRQOL, particularly experiencing higher levels of burden and effects of kidney disease. Potential explanations include sensation of thirst and dry mouth [36] from such restrictions which can cause distress [37,38] and induce difficulty in adhering to prescribed fluid restrictions [39] further exacerbating excessive fluid states and unpleasant symptoms associated with this (e.g., shortness of breath and oedema). Patients often perceive prescribed fluid restrictions as burdensome, and as contributing to anxiety, fatigue, and limitations in daily functioning and social engagement [40,41]. A Brazilian study of patients on HD with negligible or absent urine output (≤200 mL/day), who were less accepting of their fluid restriction, had poorer HRQOL outcomes compared to those who were more accepting of their fluid restriction [29]. This may in part be due to less aggressive fluid restriction prescriptions for those who have higher residual urine volume and consequent capacity to have better management of their fluid balance, removal of uremic toxins, and control of electrolytes through this urinary route compared to people who have less residual urinary function [42,43]. Although our study showed no difference in level of acceptance of fluid and diet restriction between people with residual urine output > 200 mL/day and those ≤200 mL/day (*n* = 6) in the full study cohort (replicated in our HD only cohort), our study numbers may not be large enough to detect a difference between these two groups. Furthermore, renal diets are complex and difficult to navigate for people with CKD who are already faced with socio-economic challenges and a burden of chronic disease [44,45]. These factors compound psychosocial distress and lower emotional well-being, making understanding and acceptance of, and adherence to, diet and fluid restrictions more challenging for the CKD cohort [46].

Interventions to increase acceptance and adherence to fluid and diet restriction can be implemented with benefits to HRQOL and fluid management outcomes. These include education, cognitive, and behavioral strategies to enhance self-management of fluid consumption, which have been shown to improve fluid restriction adherence with no adverse effects on psychosocial function [47]. Individual-based cognitive behavioral therapies may improve HRQOL and markers of fluid status in people on HD [48] and PD [49]. Motivational interviewing has also been reported to improve sodium intake and urinary sodium excretion [23,24]. This highlights how addressing issues with emotional well-being can be important to patient perception of and adherence to fluid and dietary advice. Technological adjuncts, including text/short messaging service/application-messages, in addition to face-to-face delivery can enhance accessibility to dietetic education and support in a timely manner [50]. Use of symptom-augmenting adjuncts such as chewing gum and saliva substitutes may alleviate thirst and reduce xerostomia [51]. Addressing HRQOL with consideration of body sodium measures and patient acceptance of diet and fluid restrictions could improve the patient experience of kidney disease, which is inherently linked to clinical outcomes including mortality and hospitalization [52,53].

We acknowledge some limitations of this study. Self-reported data was collected, including for 24 h dietary sodium intake, which may lead to recall bias. Binary classification in this study of “lower level of acceptance” versus “higher level of acceptance” of fluid and diet restrictions was used as this was a cross-sectional pilot study with small numbers; and such categorization may mask the effects of gradations in acceptance. Future studies with larger numbers could evaluate differences in gradations further. The small participant numbers may also limit generalizability. Although associations were identified, the observational nature meant we could not restrict dietary sodium intake or assess adherence to prescribed dietary and sodium restrictions. Future studies could explore relationships between acceptance of and adherence to such clinician-prescribed restrictions. There may be residual confounding factors such as socio-cultural economic practices and accessibility to healthcare therapies that could affect HRQOL that were not explored in this study. However, to our knowledge, this is the first study to explore differences between dietary sodium intake and urinary sodium excretion measures and HRQOL in people across the spectrum of CKD.

## 5. Conclusions

People with CKD with low 24 h urinary sodium excretion and lower acceptance of diet and fluid restriction have poorer HRQOL in kidney-disease specific domains. Further studies exploring the underlying mechanism between urinary sodium excretion and HRQOL in CKD are needed. Diet and fluid management in CKD is typically focused on reinforcement of restrictions to achieve clinical outcomes and biochemical targets; however, more attention should be directed to assessing, understanding, and addressing patient acceptance of these restrictions. Concurrent HRQOL, dietary sodium intake, and urinary excretion assessments, and adoption of strategies to enhance patient acceptance of fluid and diet restrictions, could empower both healthcare providers and patients to improve HRQOL and clinical outcomes in people with CKD.

## Figures and Tables

**Table 1:** Demographics, co-morbidities, and medications of participants.

	CKD (*n* = 28)	HD (*n* = 13)	PD (*n* = 12)	All (*n* = 53)	*p*-Value
Age (years), median (IQR)	56 (46.5–65.5)	68 (49–78)	62.5 (46.5–73.5)	60 (48–68.5)	0.17
Sex (male), *n* (%)	18 (64.29)	10 (76.92)	9 (75)	37 (69.81)	0.73
Body mass index (kg/m^2^)	26.95 (23.4–30.5)	27.5 (23.9–29.8)	27.15 (24.6–28.5)	27.3 (23.9–29.2)	0.91
eGFR (mL/min/1.73 m^2^)	30.14 ± 13.25	N/A	N/A		
Dialysis vintage (days)	N/A	596 (426–1411)	396 (160.5–728.3)	521(292–867)	0.06
Etiology of kidney disease *n* (%)					
Hypertension	0	2 (15.4)	2 (16.7)	4 (7.6)	
Type-2 diabetes	7 (25)	4 (30.8)	2 (16.7)	13 (24.5)	
IgA Nephropathy	7 (25)	2 (15.4)	1 (8.3)	10 (18.9)	
Polycystic kidney disease	4 (14.3)	0	2 (16.6)	6 (11.3)	
Other	10 (35.7)	5 (38.5)	5 (41.7)	20 (37.7)	
Co-morbidities (*n*, %)					
Hypertension	17 (60.7)	11 (84.6)	11 (91.7)	39 (73.6)	0.09
Diabetes	7 (25)	5 (38.5)	3 (25)	15 (28.3)	0.72
Peripheral vascular disease	0	0	0	0	
Coronary artery disease	1 (3.6)	6 (46.2)	1 (8.3)	8 (15.1)	0.003
Congestive heart failure	0	3 (23.1)	0	3 (5.7)	<0.001
Cerebrovascular disease	1 (3.6)	1 (7.7)	2 (16.7)	4 (7.6)	0.24
Medications (*n*, %)					
ACEi/ARB	18 (64.3)	3 (33)	9 (75)	30 (56.6)	0.17
Beta-blocker	7 (25)	2 (22)	3 (25)	12 (22.6)	1
Calcium channel blocker	11 (39.3)	3 (33)	3 (25)	17 (32.1)	0.75
Diuretic	8 (28.6)	4 (44)	8 (66.7)	20 (37.7)	0.08
SGLT2-i	7 (25)	N/A	N/A	7 (13.2)	

eGFR: estimated glomerular filtration rate; ACEi: angiotensin-converting enzyme inhibitor; ARB: angiotensin receptor blocker; and SGLT2-i: sodium/glucose cotransporter 2 inhibitor.

**Table 2:** Sodium and volume measures of participants.

	CKD (*n* = 28)	HD (*n* = 13)	PD (*n* = 12)	All (*n* = 53)	*p*-Value
Scored sodium questionnaire (points), [median (IQR)] ^a^	84.75 (64.8–99.8)	59 (53–80)	54 (29–74.6)	71.75 (47.4–85.8)	0.02
24 h dietary sodium record (mg) ^b^	1980.57 (1016.1–2906.8)	1495.5 (1186.2–1684.5)	2663.23 (1296.4–4197.9)	1649.58 (1093.1–3094.8)	0.472
24 h urine volume (mL) ^c^	2353 (1810–2830)	550 (350–1230)	1300 (820–1730)	1900 (1330–2600)	<0.001
24 h urine sodium (mmol/L) ^d^	60 (40.5–91.3)	47 (34–87)	57 (46–73)	57.5 (43–80.8)	0.888
24 h urine sodium excretion (mg/day) ^d^	3043.59 (2290.5–4564.1)	912.46 (379.3–1427.7)	1704.3 (1131.6–2747.5)	2479.4 (1409.7–3497.1)	<0.001
Random spot urine sodium (mmol/L) ^e^	56 (40–71.5)	29 (20–51)	45 (40.5–80)	51.5 (36.75–70.5)	0.151
Serum sodium (mmol/L)	138 (137–140)	137 (135–139)	130 (125–140)	138 (136–140)	0.288
Office systolic blood pressure (mmHg)	125 (120–140)	135 (116–154.5)	130 (125–140)	130 (120–140)	0.198

^a^ data available for 21 CKD, 11 HD, and 12 PD participants. ^b^ data available for 25 CKD, 13 HD, and 10 PD participants. ^c^ data available for 27 CKD, 5 HD, and 11 PD participants. ^d^ data available for 26 CKD, 5 HD, and 11 PD participants. ^e^ data available for 28 CKD, 7 HD, and 11 PD participants.

**Table 3:** KDQOL-36 components stratified by CKD group.

KDQOL-36 Component Scores	CKD (*n* = 28)	HD (*n* = 13)	PD (*n* = 12)	All	*p*-Value
SF physical composite score ^a^ [median (IQR)]	44.0 (31.4–51.3)	39.9 (21.7–48.3)	37.9 (35.1–51.5)	40.7 (32.1–49.0)	0.47
SF mental composite score ^a^	45 (35.5–56.5)	51.8 (37.1–59.0)	51.7 (45.7–57.4)	50.1 (37.3–57.4)	0.73
Burden of kidney disease	68.7 (39.1–87.5)	43.8 (28.1–59.4)	50 (31.3–60.9)	50 (31.3–78.1)	0.03
Symptoms of kidney disease	75 (68.8–89.8)	79.2 (66.7–93.5)	88.5 (71.9–89.6)	81.8 (70.3–90.3)	0.67
Effects of kidney disease	86.6 (71.9–93.8)	65.6 (29.7–85.9)	75 (60.2–82.8)	78.1 (60.9–90.6)	0.05

^a^ data available for 25 CKD, 11 HD, and 11 PD.

**Table 4:** (**A**) KDQOL-36 stratified by high vs. low dietary scored sodium questionnaire. (**B**) KDQOL-36 stratified by high vs. low 24 h dietary record. (**C**) KDQOL-36 stratified by high vs. low 24 h urinary sodium excretion.

(A)			
Dietary SSQ Points			
	Low (<65 Points)	High (≥65 Points)	*p*-Value
Sodium level–no./total, (%)	19/44 (43.2)	25/44 (56.8)	
KDQOL-36 component scores			
SF physical composite score, [median (IQR))]	40.0 (33.7–51)	40.3 (33.7–49.0)	0.66
SF mental composite score	50.0 (37.3–55.3)	51.7 (43.6–58.5)	0.43
Burden of kidney disease	43.8 (31.3–81.3)	50 (28.1–75)	0.66
Symptoms of kidney disease	86.4 (70.8–93.2)	83.3 (70.5–90.3)	0.73
Effects of kidney disease	84.4 (59.4–87.5)	78.1 (62.5–88.2)	0.8
**(B)**			
**24 h Dietary Record**			
	**Low (<2.3 g/Day Sodium Intake)**	**High (≥2.3 g/Day Sodium Intake)**	***p*-Value**
Sodium level–no./total no. (%)	31/48 (64.6)	17/48 (35.4)	
KDQOL-36 component scores			
SF physical composite score, [median (IQR)]	40.5 (33.3–48.7)	41.0 (32.3–47.9)	0.84
SF mental composite score	51.74 (41–58.5)	49.0 (36.7–57.4)	0.52
Burden of kidney disease	56.3 (31.3–81.3)	50 (34.4–87.5)	0.85
Symptoms of kidney disease	84.1 (70.5–93.2)	75 (66.5–92.0)	0.87
Effects of kidney disease, (mean ± SD)	73.1 ± 23.1	70.4 ± 27.1	0.88
**(C)**			
**24 h Urinary Sodium Excretion**			
	**Low (<2.3 g/Day Urinary Sodium Excretion)**	**High (≥2.3 g/Day Urinary Sodium Excretion)**	***p*-Value**
Sodium level–no./total no. (%)	17/42 (40.5)	25/42 (59.5)	
KDQOL-36 component scores			
SF physical composite score, [median (IQR)]	40.2 (37.2–45.4)	43.9 (31.4–55.0)	0.49
SF mental composite score	45.9 (37.3–56.3)	50.7 (38.4–57.4)	0.79
Burden of kidney disease	50 (28.1–65.6)	68.8 (43.8–87.5)	0.06
Symptoms of kidney disease	72.7 (62.5–88.5)	86.4 (73.9–93.2)	0.06
Effects of kidney disease	71.4 (54.7–85.1)	87.5 (71.9–93.8)	0.03

**Table 5:** (**A**) KDQOL-36 scores stratified by level of acceptance of diet restriction. (**B**) KDQOL-36 scores stratified by level of acceptance of fluid restriction.

(A)			
Acceptance of Diet Restriction			
	Higher Acceptance (Classified as Not at All Bothered or Somewhat Bothered)	Lower Acceptance (Classified as Moderately to Extremely Bothered)	*p*-Value
no./total no. (%)	40/53 (75.5)	13/53 (24.5)	
Physical composite score	43.2 (32.5–52.6)	39.0 (30.8–43.4)	0.18
Mental composite score	51.5 (40.9–57.7)	41.7 (31–54.7)	0.17
Burden of kidney disease	62.5 (43.8–85.9)	31.3 (9.4–56.3)	0.003
Symptoms of kidney disease	62.5 (43.8–85.9)	72.5 (60.8–76.0)	0.06
Effects of kidney disease ^a^	83.3 (62.5–91.7)	37.5 (27.1–75)	<0.001
**(B)**			
**Acceptance of Fluid Restriction**			
	**Higher Acceptance** **(Classified as Not at All Bothered or Somewhat Bothered)**	**Lower Acceptance** **(Classified as Moderately to Extremely Bothered)**	***p*-Value**
no./total no. (%)	43/53 (81.1)	10/53 (18.9)	
Physical composite score	43.0 [33.7–51.5]	39.5 [24.2–42.8]	0.16
Mental composite score	51.3 [40.6–57.5]	40.3 [32.5–55.1]	0.35
Burden of kidney disease	62.5 [43.8–87.5]	31.3 [18.8–46.9]	0.002
Symptoms of kidney disease	84.1 [70.5–89.6]	73.9 [65.1–94.3]	0.67
Effects of kidney disease ^a^	83.3 [62.5–91.7]	52.1 [28.1–66.5]	<0.001

^a^ corrected for diet and fluid restriction which are components of these scales.

## Data Availability

The data underlying this article will be shared on reasonable request to the corresponding author. The data are not publicly available due to privacy reasons.
